# Physicians' Perspective on a Multidisciplinary Approach to Dysphagia Management

**Published:** 2019-05

**Authors:** Jalal Bakhtiyari, Raheb Ghorbani, Masoomeh Salmani, Mozhgan Asadi, Sadaf Irani, Rana Esmaeel Abadi

**Affiliations:** 1 *Neuromuscular Rehabilitation Research* *Center, Semnan University of Medical Sciences, Semnan, Iran.*; 2 *Social Determinants of Health Research Center, Semnan University of Medical Sciences, Semnan, Iran.*; 3 *Department of Speech and Language Therapy, Rehabilitation Faculty, Semnan University of Medical Sciences, Semnan, Iran.*; 4 *Student Research Committee, Semnan University of Medical Sciences, Semnan, Iran.*

**Keywords:** Attitude, Dysphagia, Deglutition disorders, Knowledge

## Abstract

**Introduction:**

Professionals need a multidisciplinary approach to manage oropharyngeal dysphagia (OPD). Each team member should be aware of the signs and symptoms of OPD and collaborate with other team members to reach an optimum outcome. This study aimed to evaluate the knowledge and attitude of Iranian physicians regarding dysphagia and speech and language therapy roles in the management of dysphagia.

**Materials and Methods:**

In this nonexperimental descriptive study, 133 physicians filled a researcher-made questionnaire entailing three sections, each of which recorded the participants’ demographic information, OPD knowledge (23 items), and attitude toward OPD (5 items).

**Results:**

Iranian physicians correctly answered 70.3% of the questions indicating that they had a moderate level of knowledge on OPD. However, only 53% of the physicians referred their patients to speech-language pathologists, and almost 50% of them reported a significant recovery after speech therapy in their patients.

**Conclusion:**

It seems that Iranian physicians need to adopt an interdisciplinary approach to manage OPD. Achievement of sufficient knowledge on the pathology of OPD, along with collaboration with other team members, can improve the outcome for patients with OPD.

## Introduction

Swallowing disorder or oropharyngeal dysphagia (OPD) is a common symptom in different medical conditions, such as stroke, Parkinson's disease, multiple sclerosis, and problems affecting the head and neck, including cancers and surgeries (1). Even though OPD can occur in different age groups, it is more common among the geriatric population (2). 

The side effects of OPD turn it into a complicated condition which needs immediate and prompt evaluations and interventions. Malnutrition and dehydration, aspiration pneumonia, compromised general health, chronic renal disease, and choking are among the side effects that can endanger the patient's life (1,3,4). The OPD can increase the responsibilities of the professionals and families due to its consequences (5,6). 

There are a number of studies reporting the prevalence of dysphagia in Iran. For instance, in a study conducted by Barikroo et al., 41.2% of the elderly individuals living in nursing home centers were reported to have OPD (7). Furthermore, in a couple of studies, the prevalence of OPD was estimated at 31.7% in patients with multiple sclerosis and 55% in acute stroke patients (8,9). However, in other medical conditions, such as Parkinson’s disease, dementia, and head and neck cancers, the prevalence of dysphagia is unclear.

Early assessment and intervention probably could be helpful in decreasing the patients' problems. However, this cannot be easily accomplished because of the different problems these patients are struggling with. A multidisciplinary approach should be undertaken to implement the right treatment for these patients. In a multidisciplinary approach, a team, consisting of a physician, nurses, a speech-language pathologist, a nutritionist, patient's family, and other related professionals, will collaborate to provide the best services for the patients (1,4,10). 

To accomplish this end, each team member needs to be aware of the patients' problems, have the right information, and be familiar with the proper assessment procedure and interventional options. In this team, speech-language pathologists have the main role throughout the process (from the identification, diagnosis, and assessment to the management of the OPD). The implementation of this approach is an ideal option to deal with OPD. However, there is a different process in Iran to manage these patients. Physicians are the first encounters who decide on the types of intervention. The data regarding the prevalence of OPD in Iran and comparison of such data with the number of referrals to the speech-language pathologists are indicative of a gap in this domain. This gap could probably be the result of the lack of knowledge about the role of speech-language pathologists, OPD, or the course of treatment. With this background in mind, the present study was conducted to find out the knowledge and attitudes of Iranian physicians regarding OPD.

## Materials and Methods

This nonexperimental descriptive study was performed in two different phases. 

*Phase 1: Questionnaire Development*

In Iran, speech-language pathologists do not have any standard questionnaires to evaluate physicians' knowledge and attitude regarding dysphagia. In the review of the literature, the first author did not find any questionnaires in languages other than Persian. Therefore, a questionnaire was designed by the researchers based on the relevant articles. The questionnaire consisted of three different parts, recording demographic characteristics, knowledge about OPD, and physicians' attitudes toward a multidisciplinary approach to dysphagia management. The face and content validities of the questionnaire were evaluated. To this end, six experts (i.e., three speech-language pathologists experienced in dysphagia management, a neurologist, an internist, and an otorhinolaryngologist) blind to the present study were invited to review the preliminary version through electronic mails. 

The experts evaluated the clarity, concept, understandability, and grammaticality of each question based on a 5-point Likert scale. The reliability of the questionnaire was investigated using the test-retest method. In this regard, 10 physicians were randomly selected and asked to complete the questionnaire twice within a 2-week interval.The final version of the questionnaire still had three parts. The first part as the demographic part covered such information as age, gender, specialty, the country in which the physician was graduated, workplace, and length of work experience. The second part, entailing 23 items, investigated the physicians' knowledge of OPD, and the third part evaluated the physicians' attitudes toward OPD in 5 items. In the knowledge section, 14 items directly targeted physicians' knowledge about the signs and symptoms of OPD. Items 1 and 11 were yes/no questions, and the remained items were multiple-choice. The scoring system was in a dichotomous manner. If the participant chose "I don't know" or gave a wrong answer, the score was zero, and a correct answer was scored "1".

**Phase 2: Participants & Procedure**

For sampling, 15 out of 60 public and private hospitals were randomly selected from three cities of Iran, namely Tehran, Karaj, and Semnan, during 2016-2017. All related specialists (i.e., general physicians,neurologists, otolaryngologists, pulmonologists, and internists) were invited through meetings.

A total of 250 physicians agreed to be part of this study; however, 133 physicians filled out the questionnaire. This study was approved by the Research Ethics Committee of Semnan University of Medical Sciences, Semnan, Iran (reference No. IR.SEMUMS.REC.1395.63). 

**Statistical Analysis**

Statistical analysis was performed using SPSS software (version 21). P-value less than 0.05 was considered statistically significant. Face and content validities were analyzed by calculating the mean scores of the rating of each item. Internal consistency of the questionnaire was examined using Cronbach’s alpha coefficient. In addition, the test-retest reliability of the scale was evaluated using the intraclass correlation coefficient. The data were presented as mean and standard deviation. Additionally, the Shapiro-Wilk, Mann-Whitney U, Kruskal-Wallis, and Pearson tests, as well as ANOVA, were applied to analyze the data. 

## Results

**Questionnaire Evaluation**

After making the necessary corrections to improve the understandability and grammaticality of the questionnaire, the face validity of the instrument was approved according to the scores assigned by the expert panel. Furthermore, the content validity ratio and content validity index were obtained at 99% or above for all items on the basis of Lawshe's method. Accordingly, the questionnaire was concluded to have good content validity. Test-retest reliability suggested that the questionnaire scores were stable over time. In this regard, the achievement of a p-value less than 0.001 indicated that the questionnaire had excellent reliability. In addition, the Cronbach’s alpha coefficient was estimated at 0.88. The intraclass correlation coefficient for all items was within a range of 0.95-0.98.

**Demographic Characteristics**

The present study evaluated the knowledge and attitude of 133 physicians regarding OPD. A researcher-made questionnaire was the main tool to collect data. Table 1 presents some general information about the participants. Half of the physicians had more than five years of work experience. The majority of the subjects were graduated from public and well-known universities. This factor could have some effects on the physician's knowledge regarding the issue under investigation. Education in public schools provides the physicians with more opportunities to earn experience on different subjects and in this case OPD. The mean age of the physicians participating in this study was 41.5±10.3 years.

**Table1:** Demographic characteristics of participants (n=133).

**Participants characteristics **	**n (%), Mean (SD)**
Gender	
Male Female	93 (69.9%) 40 (30.1%)
Age	41.5±10.3
Specialty	
Neurologist Otolaryngologist Internist General practitioner Others	32 (24.1%) 14 (10.5) 48 (36.1%) 25 (18.1%) 14 (10.5%)
Place of graduation	
Public universities Azad universities	127 (95.5%) 6 (4.5%)
Length of work experience	
>1 1-3 3-5 ≥5	18 (13.5%) 9 (6.8%) 36 (27.1%) 70 (52.6%)

**Knowledge on Dysphagia**

Table 2 presents the scores of physicians' knowledge on OPD in two parts. The first score includes all 23 items related to the physicians' knowledge on OPD to provide a general picture in this regard. The second score is related to 14 items evaluating the knowledge on OPD signs and symptoms. This score could be a rough estimation of the specific knowledge on this subject. The knowledge section with 23 items gave a perspective on physicians' knowledge about OPD. The maximum and minimum scores in this section were 23 and 0, respectively (Table.2). The mean total score of knowledge was 15.5±3.2 (range: 3-22). 

Three levels of knowledge were defined based on the physicians’ scores. Correct responses to less than < 50%, 50-75%, and > 75% of the items represented weak, moderate, and good levels of knowledge about OPD, respectively. Iranian physicians answered 70.3% of the questions correctly denoting that they had a moderate level of knowledge about OPD.

**Table 2:** Physicians' knowledge on oropharyngeal dysphagia (n=133)

**Issues of knowledge**	**Minimum**	**Maximum**	**Mean**	**Std. Deviation**
Total knowledge (23 items)	2	23	15.5	3.2
Signs and symptoms knowledge (14 items)	0	14	9.6	2.2

The mean correctly answered items evaluating knowledge on the signs and symptoms of OPD was 9.6±2.2 out of 14 (range: 0-14).

Table 3 lists the percentage of correct answers for each item.The physicians had a good level of knowledge about the swallowing mechanism, signs, symptoms, and complications of OPD, as well as its evaluation process. According to results, 84% of the participants reported to have at least one patient with OPD. These physicians were mainly neurologists, otorhinolaryngologists, internists, and pulmonologists.

**Table 3:** Physicians' knowledge of sign and symptoms of oropharyngeal dysphagia (n=133)

**Signs and symptoms**	**Correct response**	
	n	%
Choking and/or throat clearing	98	73.3
Vomiting	111	83.5
Reduced pharyngeal reflex (gag)	104	78.2
Multiple swallows per bolus	86	64.7
Frequent temperature spikes after eating	125	94
Aspiration and penetration	58	43.6
Chest pain	67	50.4
Hoarse voice and changed voice	52	39.9
Reduced laryngeal elevation	70	52.6
Food remaining in the mouth	43	32.2
Skin irritations	64	48.1
Poor movement of the tongue	47	35.3
Difficulty initiating swallowing	104	78.2
Drooling	42	31.5

However, only 53% of the participants were aware that the speech-language pathologists are responsible for the evaluation of these patients and the associated intervention process and referred their patients to these specialists. Among these specialists, neurologists had a higher percentage for referring the patients to the speech-language pathologists than the other specialists (Table.4). 

The results indicated no significant relationship between physicians' knowledge and different factors, including age, gender, workplace, and work experience (P≥0.05). However, there was a significant relationship between the physicians' specialty and level of knowledge about OPD (Table.4).

**Table 4:** Relationship of physicians' knowledge with different factors

	**r**	**P-value**	**r**	**P-value**	**P-value (Kruskal-Wallis)**		
	**Pearson**						
Physicians' knowledge of OPD signs	0.128	0.143	0.111	0.204	0.729	0.107	<0.001
Physicians' knowledge Total	0.051	0.559	0.082	0.346	0.495	0.131	<0.001

OPD: oropharyngeal dysphagia

*Attitude toward oropharyngeal dysphagia*

The physicians' attitudes regarding OPD were evaluated in seven items. Each item was answered on a 5-point Likert scale from completely agree (scored 5) to completely disagree (scored 1). Three different types of attitudes (i.e., positive, neutral, and negative) could be inferred from these items. The highest and lowest scores in this part were 35 and 7, respectively. In this part, scores of < 17.5, 17.5-26.25, and 26.25 were indicative of negative, moderate or neutral, and positive attitudes, respectively. In this classification, only 46.8% of the participants had a positive attitude, and the remaining scores were below 17.5. 

## Discussion

The present study aimed to evaluate the knowledge and attitude of Iranian physicians regarding OPD. The results of this study indicated that physicians in Iran had a low level of knowledge regarding the role of speech-language pathologists in the management of OPD. In addition, these physicians had a negative attitude toward managing OPD by speech-language pathologists. 

This finding is in contrast with the findings presented by Anderle et al. investigating the knowledge of the Brazilian medical and nursing teams about the management of oral medications in hospitalized adult dysphagic patients (11). They found out that the teams were completely aware of dysphagia and speech-language pathologists’ roles during the intervention and assessment process. This difference might be related to the scope of practice and employment areas of speech-language pathologists in different countries. 

In Iran, most of the speech-language pathologists are employed by the Ministry of Education to work at schools, aged care systems, rehabilitation centers, and private sectors. There are a few Iranian speech-language pathologists working at hospitals or collaborating as a part of the medical teams. However, in Brazil, these pathologists are mainly active in the public health domain, and oral myology is one of their treatment focus areas. 

The other issue that can explain the situation in Iran could be the swallowing disorders field for speech-language pathologists. The first paper and textbook on dysphagia rehabilitation were published in 1972 and 1983 (5,6), respectively. In addition, the first guideline in this regard was released by the American Speech-Language-Hearing Association in 1987. Nonetheless, in Iran, the teaching of the first formal curriculum regarding dysphagia began in 2006 in the Departments of Speech and Language Therapy. Accordingly, it is not surprising that the first PhD dissertation on dysphagia was performed in 2014. 

Iranian physicians' knowledge about the signs and symptoms of OPD was at a relatively good level. This could be related to the textbook materials. The significant relationship between the physicians' specialty and their knowledge could probably explain this finding. However, in a Brazilian study, it was reported that their medical teams did not have enough knowledge on the signs and symptoms of swallowing disorders. Furthermore, their knowledge about the alternative therapies (i.e., treatments other than medications) was not at a sufficient level. This could be also the result of educational curriculum in each country. 

The investigation of the available database showed that the participants of the majority of the studies in this field were nurses (12-15). This might be due to the difference in the health service delivery across countries. In Iran, physicians are in charge of helping patients to make decisions about their treatment procedures. Nonetheless, in other countries, there is a medical team assisting patients during the evaluation and intervention process. 

One of the limitations of the present study was that it failed to include all the regions of Iran. The situation might be different in other cities of Iran. In addition, we did not include all medical professionals, such as Intensive Care Unit specialists, pulmonologists, surgeons, and related professions. Therefore, other studies with a wider coverage might reach a more comprehensive picture.

## Conclusion

In summary, Iranian physicians were fairly familiar with dysphagia and its signs and symptoms. They had insufficient knowledge on the other team members' roles in OPD management. Regarding to the importance of swallowing disorders and their consequences in different health conditions and the importance of early diagnosis and early intervention, any workshops and educational courses targeted toward the enhancement of physicians’ knowledge and awareness regarding a team approach would be ideal. Implementation of continuous educational programs and collaboration with other team members could be regarded as solutions to increase the patients’ chance to receive a better treatment. 

Future studies should investigate the factors that affect the physician's perspective on teamwork to manage OPD. 

**Compliance with Ethical Standards**

This study was funded by the Student Research Committee of Semnan University of Medical Sciences, Semnan, Iran (grant No. 1069). 

**Ethical Approval**

This study was approved by the Human Rights Committee of Semnan University of Medical Sciences (Reference No. 63 IR.SEMUMS.REC.1395-63).Informed consent was obtained from all participants included in the study.

## References

[1] Logemann JA (1998). Evaluation and Treatment of Swallowing Disorders.

[2] Sura L, Madhavan A, Carnaby G, Crary MA (2012). Dysphagia in the elderly: management and nutritional considerations. Clinical Interventions in Aging.

[3] Grohe ME, Crary MA (2015). Dysphagia: Clinical Management in Adults and Children.

[4] Leonard R, Kendall K (2008). Dysphagia Assessment and Treatment Planning: A Team Approach.

[5] Vesey S (2013). Dysphagia and quality of life. British Journal of Community Nursing.

[6] Davis LA (2007). Quality of Life Issues Related to Dysphagia. Topics in Geriatric Rehabilitation.

[7] Barikroo A, Hosseini Z, Ansari Z (2012). The prevalence of oropharyngeal dysphagia among nursing home residents in Isfahan. Journal of Research in Rehabilitation Science.

[8] Poorjavad M, Derakhshandeh F, Etemadifar M, Soleymani B, Minagar A, Maghzi A-H (2010). Oropharyngeal dysphagia in multiple sclerosis. Multiple Sclerosis Journal.

[9] Bakhtiyari J, Sarraf P, Nakhostin-Ansari N, Tafakhori A, Logemann J, Faghihzadeh S (2015). Effects of early intervention of swallowing therapy on recovery from dysphagia following stroke. Iranian Journal of Neurology.

[10] Huckabee ML, Pelletier CA (1999). Management of Adult Neurogenic Dysphagia.

[11] Anderle P, Rech RS, Pasqualeto VM, Goulart BNGd (2018). Conhecimento das equipes médicas e de enfermagem sobre o manejo de medicamentos orais no paciente adulto disfágico hospitalizado. Audiology - Communication Research.

[12] Morphet J, Clarke AB, Bloomer MJ (2016). Intensive care nurses' knowledge of enteral nutrition: A descriptive questionnaire. Intensive Crit Care Nurs.

[13] McCullough KC, Estes JL, McCullough GH, Rainey J (2007). RN Compliance With SLP Dysphagia Recommendations in Acute Care. Topics in Geriatric Rehabilitation.

[14] Albini RMN, Soares VMN, Wolf AE, Gonçalves CGdO (2013). Conhecimento da enfermagem sobre cuidados a pacientes disfágicos internados em unidade de terapia intensiva. Revista CEFAC.

[15] Durgude Y, Cocks N (2011). Nurses' knowledge of the provision of oral care for patients with dysphagia. Br J Community Nurs.

